# Hyaluronan- and RNA-binding deubiquitinating enzymes of USP17 family members associated with cell viability

**DOI:** 10.1186/1471-2164-7-292

**Published:** 2006-11-16

**Authors:** Ju-Mi Shin, Kyong-Jai Yoo, Myung-Sun Kim, Dongku Kim, Kwang-Hyun Baek

**Affiliations:** 1 Graduate School of Life Science and Biotechnology, Cell and Gene Therapy Research Institute, Pochon CHA University, CHA General Hospital, Seoul 135-081, Korea

## Abstract

**Background:**

Protein degradation by the ubiquitin system plays a crucial role in numerous cellular signaling pathways. Deubiquitination, a reversal of ubiquitination, has been recognized as an important regulatory step in the ubiquitin-dependent degradation pathway.

**Results:**

While identifying putative ubiquitin specific protease (USP) enzymes that contain a conserved Asp (I) domain in humans, 4 *USP17 *subfamily members, highly homologous to DUB-3, have been found (USP17K, USP17L, USP17M, and USP17N), from human chorionic villi. Expression analysis showed that *USP17 *transcripts are highly expressed in the heart, liver, and pancreas and are expressed moderately in various human cancerous cell lines. Amino acid sequence analysis revealed that they contain the highly conserved Cys, His, and Asp domains which are responsible for the deubiquitinating activity. Biochemical enzyme assays indicated that they have deubiquitinating activity. Interestingly, the sequence analysis showed that these proteins, with exception of USP17N, contain the putative hyaluronan/RNA binding motifs, and cetylpyridinium chloride (CPC)-precipitation analysis confirmed the association between these proteins and intracellular hyaluronan and RNA.

**Conclusion:**

Here, we report that the overexpression of these proteins, with exception of USP17N, leads to apoptosis, suggesting that the hyaluronan and RNA binding motifs in these enzymes play an important role in regulating signal transduction involved in cell death.

## Background

The post-translational modification by ubiquitin (Ub) plays an essential role for numerous cellular functions such as protein degradation, cell cycle control, transcriptional regulation, immune response, apoptosis, oncogenesis, pre-implantation, and intracellular signaling pathways [1-6]. Conjugation of ubiquitin to a target protein is achieved by the sequential enzymatic actions via ubiquitin-activating enzymes (E1), ubiquitin-conjugating enzymes (E2), and ubiquitin ligases (E3). A novel ubiquitination factor (E4) required for efficient multiubiquitination has been identified in yeast [7]. Proteins modified with a polyubiquitin chain are then unfolded and degraded by the 26S proteasome [1,4,5]. Deubiquitination, a removal of ubiquitin from ubiquitin-conjugated protein substrates, is mediated by a number of deubiquitinating enzymes. Most deubiquitinating (DUB) enzymes are cysteine proteases and consist of at least five families, the ubiquitin C-terminal hydrolases (UCH), the ubiquitin specific processing proteases (USP), Jab1/Pab1/MPN-domain-containing metallo-enzymes (JAMM), Otu-domain ubiquitin aldehyde-binding proteins (OTU), and Ataxin-3/Josephin [8-10]. The USP family members vary in size and structural complexity, but all contain 6 characteristic conserved homology domains [5]. The UCH family members are relatively small molecules, which hydrolyze C-terminal amides and esters of ubiquitin [5]. The JAMM isopeptidases are known to deneddylate cullin (the CSN-5 subunit of the COP9/signalosome) and to release ubiquitin chains from proteins targeted for degradation (the RPN11 subunit of the proteasome) [11]. The members of OTU family have been reported as highly specific Ub isopeptidases, but they have no sequence homology to known DUBs [12]. Lastly, Ataxin-3 has a domain called the Josephin domain, cleaves ubiquitin-AMC, and binds to the DUB inhibitor ubiquitin aldehyde [8].

Hyaluronan is a kind of glycosaminoglycan, which is involved in the regulation of cell division [13], angiogenesis [14,15], and cell motility [16]. Hyaluronan exists in extracellular and pericellular matrices and interacts with various kinds of proteins [17]. Recent reports showed the existence of a few intracellular hyaluronan binding proteins (IHABPs) and one of them, receptor form hyaluronan-mediated motility (RHAMM) binds intracellular hyaluronan and plays multiple roles including cell cycle arrest [18] and spindle pole stability [19].

*USP17 *subfamily members have been previously identified [20] and one of them, *DUB-3*, has shown that the constitutive expression of *DUB-3 *blocks proliferation and can lead to apoptosis [21]. In this study, we have identified four novel members of *USP17 *subfamily which encode a deubiquitinating enzyme, from human chorionic villi tissues. Our study demonstrates for the first time that USP17 subfamily members of the DUB enzyme regulate apoptosis and cell death of cancerous cells and contain putative hyaluronan and RNA binding domains.

## Results

### Cloning of *USP17 *subfamily members in human chorionic villi and embryonic carcinoma cell lines

**Figure 1:**
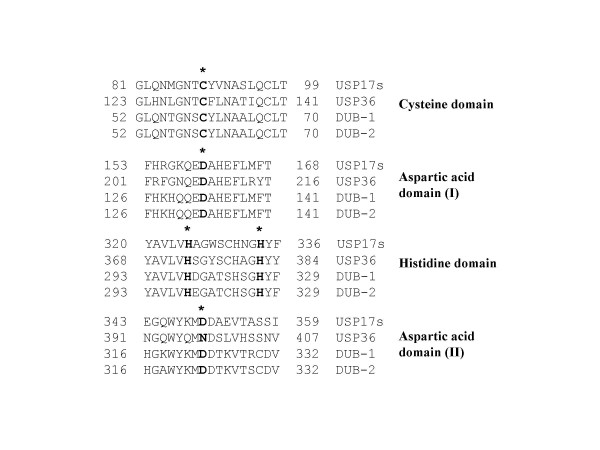
Conserved domains of USP17 subfamily members were compared with other deubiquitinating enzymes including human USP36, mouse DUB-1, and DUB-2. These domains are critical for the function of deubiquitinating enzymes.

**Figure 2:**
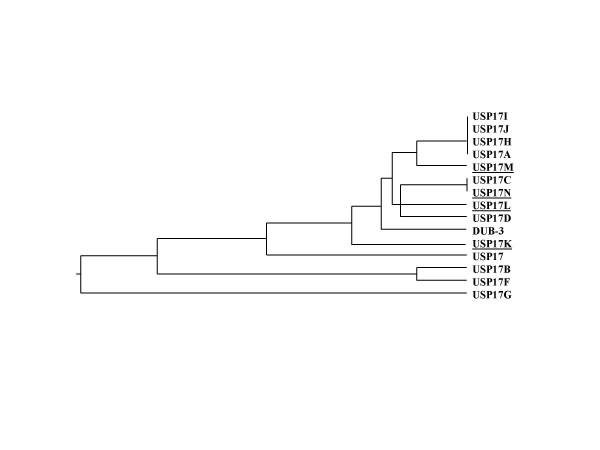
Phylogenetic tree assignments of USP17 subfamily members. Sequence names and GenBank accession numbers are as follows: USP17, AY509884; DUB-3, BC100991; USP17A, XM_377832; USP17B, XM_373239; USP17C, NM_212553; USP17D, XM_373021; USP17F, XM_376741; USP17G, XM_376754; USP17H, XM_377837; USP17I, XM_377835; USP17J, XM_377834; USP17K, AF544011; USP17L, AF544012; USP17M, AY188990, USP17N, AY533200. Novel members of USP17 subfamily are underlined. USP17E sequence identified previously [20] could not be located with the reported GenBank accession number (NM_001324).

To identify putative deubiquitinating enzymes that contain a conserved Asp (I) domain in humans, the GenBank database was accessed and searched using the BLAST algorithm at the NCBI, as described previously [22]. In this study, we obtained multiple cDNAs including *DUB-3 *[21] and *USP17 *[23] from human chorionic villi tissues and various cancer cell lines by RT-PCR. We performed RT-PCR, sequenced PCR products three times independently in order to exclude the possibility of PCR errors, and classified the genes as *USP17 *subfamily members (*USP17K*, *USP17L*, *USP17M*, and *USP17N*) due to the high homology to known sequences [20,21]. To characterize the genetic properties of human *USP17 *genes, the complete nucleotide sequences of *USP17K*, *USP17L*, *USP17M*, and *USP17N *[GenBank: AF544011, AF544012, AY188990, and AY533200, respectively] were analyzed. Nucleotide and deduced amino acid sequences were analyzed with the SeqEd V1.0.3 program and Clustal method of the MegAlign program, a multiple alignment program of the DNASTAR package. The full-length cDNAs for *USP17K*, *USP17L *and *USP17M *consist of 1593 nucleotides in open reading frame and the deduced amino acid sequence of 530 amino acids with a calculated molecular mass of 58 kDa (Additional File 1). The full-length cDNA for *USP17N *consists of 1197 nucleotides in open reading frame and the deduced amino acid sequence of 398 amino acids with a calculated molecular weight of 44.5 kDa (Additional File 1). The conserved Cys, His and Asp domains, which are thought to form the active site of the deubiquitinating enzymes, are also found in the USP17K, USP17L, USP17M and USP17N (Fig. 1); these conserved domains have been previously shown in other deubiquitinating enzymes including USP36 [24], DUB-1 [25], and DUB-2 [26]. Sequence analysis for amino acids of novel USP17 subfamily members indicates that USP17K, USP17L and USP17M have high degree of homology (>95%), and USP17N is identical with USP17C when compared with each other and USP17 family members (Table 1). Phylogenetic analysis for nucleotide sequences and deduced amino acid sequences of novel genes revealed that all novel genes were grouped into the *USP17 *subfamily (Fig. 2) and they were characterized in this report.

**Figure 3:**
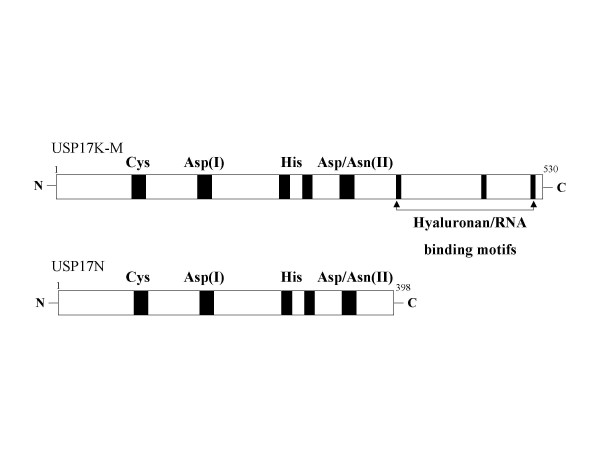
Schematic diagram of structural features for USP17 subfamily members. The domains required for deubiquitinating activity (Cys, Asp (I) His, Asp/Asn (II)) are located at the 5' end and middle of enzymes. Two potential hyaluronan binding motifs R/K(X_7_)R/K are located at amino acids 401 to 410, and 445 to 453.

Examination of the deduced amino acid sequence of these enzymes by Pfam [27] revealed that novel subfamily members of USP17 contain the putative conserved hyaluronan binding motifs (Fig. 3) -(R/K)X_7_(R/K)- [28]. These putative motifs are located between amino acid residues Arg 401 to Lys 410, and Lys 445 to Lys 453 (Fig. 3). In addition, "RGG" amino acid sequences predicted for the novel RNA binding motif are found at the C-terminus. Interestingly, USP17N lacks of these binding motifs (Fig. 3), suggesting the unique functional roles for these motifs. Even though it has been suggested that human USP17 subfamily members are homologous to murine DUB subfamily members due to the fact that they are cytokine-inducible and regulate cell growth and survival [20], hyaluronan and RNA binding motifs are not found in members of murine DUB subfamily (data not shown).

### Expression of transcripts for novel *USP17 *subfamily members in human tissues and cancerous cells

**Figure 4:**
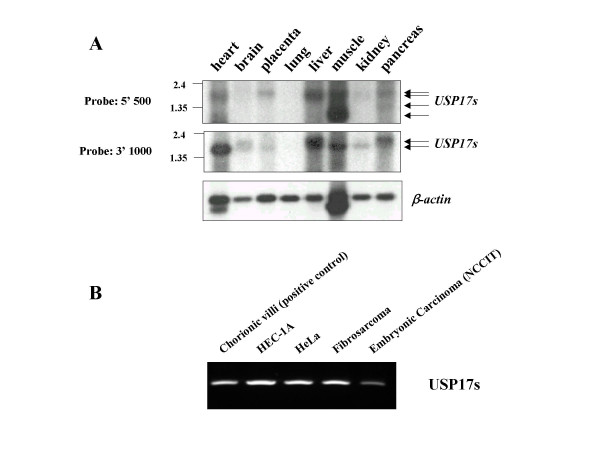
Northern blot analysis for *USP17 *subfamily members in human tissues and RT-PCR analysis in human tumor cell lines. (A) Northern blot analysis was carried out to investigate the expression level of USP17 subfamily members in human tissues. The 5' 500 bp- and 3' 1 kb-probes of *USP17L *were used. The multiple transcript sizes of *USP17 *mRNA including 1.6 kb were detected. The same blot was probed with the human β-actin as a loading control. (B) Global expression of *USP17 *subfamily members in various cancer cell lines.

To investigate the expression pattern of transcripts for novel *USP17 *subfamily members in human tissues and cancerous cells, Northern blot and RT-PCR analyses were performed. As shown in Figure 4A, Northern blot analysis revealed that transcripts for novel *USP17 *subfamily members were expressed in the heart, liver, and pancreas at a high level. Interestingly, we obtained multiple bands having different sizes (1.7 kb, 1.6 kb, 1.3 kb, 1 kb) using a 5' 500 bp probe of *USP17L *(Probe: N-500). We also detected 1.6 kb and 1.7 kb major bands of *USP17 *subfamily members using a probe designed for a 3' 1 kb probe of *USP17L *(Probe: C-1000) (Fig. 4A). It is possible that other minor mRNA transcripts around 1.3 kb and 1 kb, which have not been detected with *DUB-3 *probe [21], may be derived from alternative splicing, promoter usage, or poly (A+) usage. The presence of a few different sizes of transcript indicates that novel genes belong to the *USP17 *subfamily and that there are at least several members of this family present. In addition to various human tissues, RT-PCR analysis showed that the expression of transcripts for novel *USP17 *subfamily members was detected in various human cancer cell lines including HeLa, HEC-1A, NCCIT, and fibrosarcoma cells (Fig. 4B).

### Novel USP17 subfamily members as a deubiquitinating enzyme

**Figure 5:**
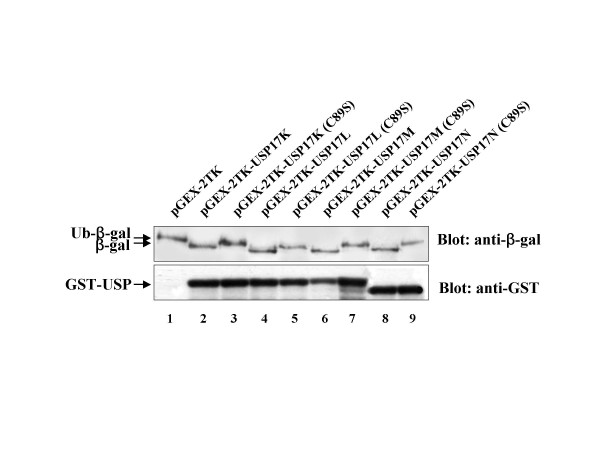
Deubiquitinating enzyme assays for novel USP17 subfamily members. *In vitro *deubiquitinating enzyme activity for USP17K, USP17L, USP17M, and USP17N. Immunoblotting analysis was performed with anti-β-gal antiserum. Coexpressed plasmids were pGEX-2TK (lane 1), pGEX-2TK-*USP17K *(lane 2), pGEX-2TK-*USP17K *(C89S) (lane 3), pGEX-2TK-*USP17L *(lane 4), pGEX-2TK-*USP17L *(C89S) (lane 5), pGEX-2TK-*USP17M *(lane 6), pGEX-2TK-*USP17M *(C89S) (lane 7), pGEX-2TK-*USP17N *(lane 8), and pGEX-2TK-*USP17N *(C89S) (lane 9). All of normal USP17 subfamily members cleaved the linkage between ubiquitin and β-galactosidase.

To determine whether novel USP17 subfamily proteins have ubiquitin specific protease enzymatic activity, deubiquitinating enzyme assays were performed. For *in vitro *enzyme assay, novel USP17 subfamily members were expressed as a GST fusion protein. As shown by immunoblotting analysis (Fig. 5), four GST fusion proteins demonstrated a strong efficient cleavage of Ub-Met-β-gal (Fig. 5; lanes 2, 4, 6, and 8). We generated the Cys conserved domain mutant for each of novel USP17 subfamily proteins (C89S) using a site-directed mutagenesis method. As expected, cells with the empty vector alone (Fig. 5, lane 1) and mutants (Fig. 5; lanes 3, 5, 7 and 9) failed to cleave Ub-Met-β-gal.

**Figure 6:**
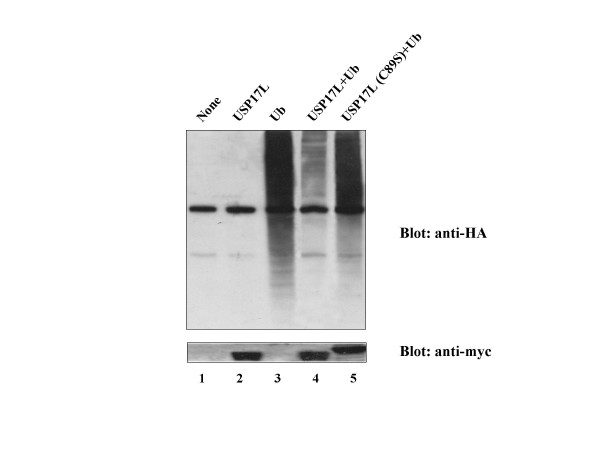
*In vivo *deubiquitinating enzyme assay for the novel USP17 subfamily member. HA-tagged pMT123-*ubiquitin *was transiently expressed in HEC-1A cells with or without pcDNA3-*myc*-*USP17L *and pcDNA3-*myc*-*USP17L *(C89S). The expression levels of polyubiquitinated proteins were decreased in cells expressing pcDNA3-*myc*-*USP17L*, but not in cells expressing pcDNA3-*myc*-*USP17L *(C89S).

For *in vivo *deubiquitinating enzyme assay, HA-tagged pMT123-ubiquitin was transiently expressed in human endometrial carcinoma cells (HEC-1A) with or without pcDNA3-myc-*USP17L *and pcDNA3-myc-*USP17L *(C89S). Then, we analyzed deubiquitinating enzyme activity by immunoblotting using a monoclonal anti-HA antibody. The expression bands of polyubiquitinated proteins were nearly invisible in cells expressing pcDNA3-myc-*USP17L *(Fig. 6; lane 4), but not in cells expressing pcDNA3-myc-*USP17L *(C89S) (Fig. 6; lane 5). The molecular weight for a mutant form of USP17L (C89S) is bigger than wild type USP17L, suggesting that there may be a conformational change due to the amino acid substitution. In addition, other USP17 subfamily members showed the deubiquitinating enzyme activity *in vivo *(data not shown). Taken together, we concluded that novel USP17 subfamily proteins have deubiquitinating enzyme activity.

### Hyaluronan/RNA-binding properties of USP17 subfamily proteins

**Figure 7:**
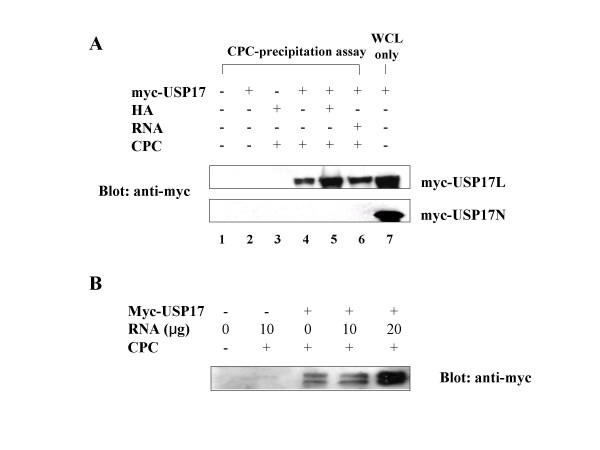
Hyaluronan/RNA-binding assays. To confirm the interaction of USP17 subfamily proteins with either hyaluronan or RNA, CPC precipitation assay was performed. Cell lysate aliquots from HeLa cells transfected with myc-tagged *USP17L *or myc-tagged *USP17N *construct were incubated with hyaluronan or RNA and subjected to CPC precipitation. (A) Coprecipitated proteins were subjected to Western blot analysis with an antibody against the myc tag. CPC precipitation without the addition of CPC or myc-tagged *USP17 *construct was used as negative controls (lanes 1–3). Without the addition of CPC, the whole cell lysate from cells transfected with myc-tagged *USP17L *or myc-tagged *USP17N *was used as a positive control (lane 7). (B) For RNA binding assay, total RNA of HeLa cells instead of hyaluronan were added.

Since it is not known whether hyaluronan-binding sites within USP17 subfamily proteins are indeed functional, we further examined whether USP17 subfamily proteins actually binds hyaluronan. The cetylpyridinium chloride (CPC)-precipitation assay has been used for investigating binding affinity [29] and the assay in our study demonstrated that USP17 subfamily proteins interact with hyaluronan based on the comparison of binding intensity between endogenous and exogenous hyaluronans (Fig. 7A, lanes 4 and 5). However, USP17N did not interact with hyaluronan (Fig. 7A, lane 5), indicating that the C-terminal domain of USP17 subfamily proteins is required for binding with hyaluronan.

Since it has been reported that the HABP4_PAI-RBP1 domain, which is involved in mRNA stability [30], interacts with both hyaluronan and RNA [17], we additionally investigated the activity of USP17 subfamily proteins for binding with RNA. We performed the CPC-precipitation assay and found that USP17 subfamily proteins also bind to RNA in a dose-dependent manner (Figs. 7A and 7B). Interestingly, USP17N did not interact with RNA as shown with hyaluronan (Fig. 7A, lane 6), indicating that the C-terminal domain of USP17 subfamily proteins is also required for binding with RNA.

### Localization study of USP17L

**Figure 8:**
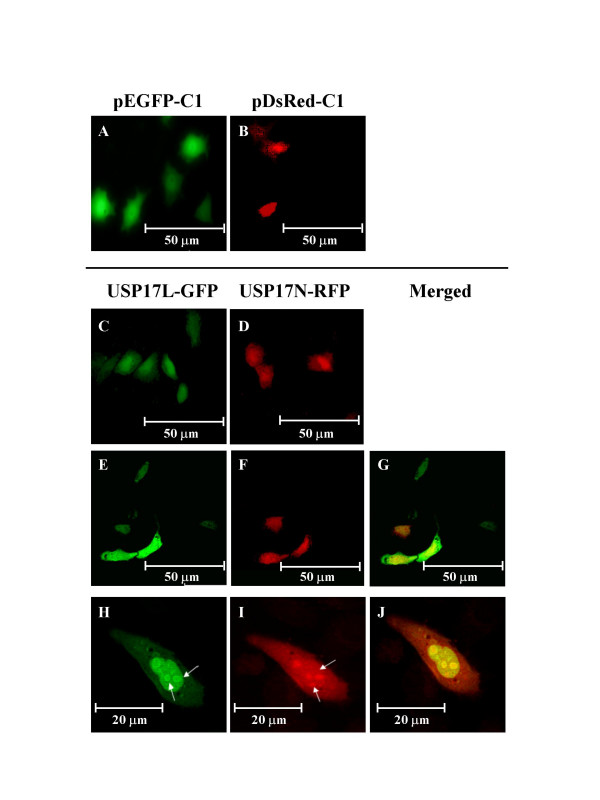
The localization of USP17L and USP17N in HeLa cells. pEGFP-C1 vector (A) or pDsRed-C1 vector (B) transfected into HeLa cells was used as controls. In addition, GFP-tagged USP17L (C) or RFP-tagged USP17N (D) transfected into HeLa cells was used as controls. (E-G) HeLa cells co-transfected with GFP-tagged USP17L and RFP-tagged USP17N are shown with lower magnification (E-G) and with higher magnification (H-J). GFP-tagged USP17L proteins are highly condensed at the nucleoli in the nucleus (H: marked by arrows). RFP-tagged USP17N proteins are present both in the nucleus and cytoplasm (I: marked by arrows). GFP-tagged USP17L and RFP-tagged USP17N were combined in HeLa cells (G and J).

In order to investigate the intracellular localization of USP17 subfamily proteins, the GFP-tagged USP17L or RFP-tagged USP17N, or both were transfected into HeLa cells. Intriguingly, USP17L proteins are found in the nucleus and are concentrated at the nucleoli (Figs. 8E and 8H) similar to intracellular hyaluronan [31]. However, USP17N proteins are localized both in the nucleus and cytoplasm (Figs. 8F and 8I). A merged format shows that USP17L and USP17N are localized in different regions (Figs. 8G and 8J). After 48 hours of transfection, cells expressing GFP-tagged USP17L showed the onset of irregular nuclear division and irregular morphology of overall cell shape compared with controls (data not shown). In contrast, cells expressing RFP-tagged *USP17N *did not show any sign of apoptosis, suggesting that hyaluronan binding ability and the localization of USP17 subfamily proteins may be critical for regulating apoptosis leading to cell death.

### Regulation of cell viability

**Figure 9:**
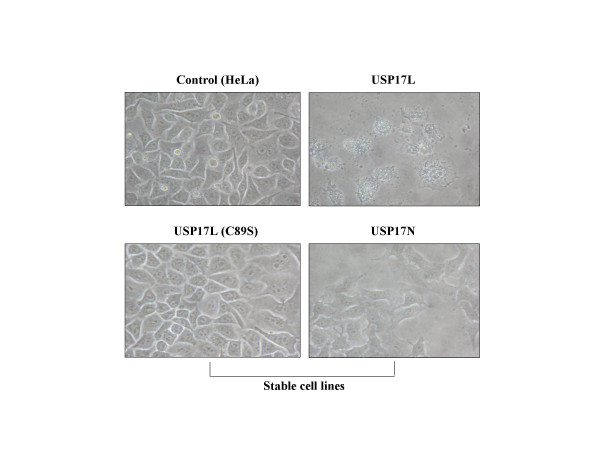
The effect of *USP17 *expression in HeLa cells. Overexpression of *USP17L*-induced apoptosis of cervical cancer cells. Either *USP17L *(C89S) or *USP17N *did not induce apoptosis.

**Figure 10:**
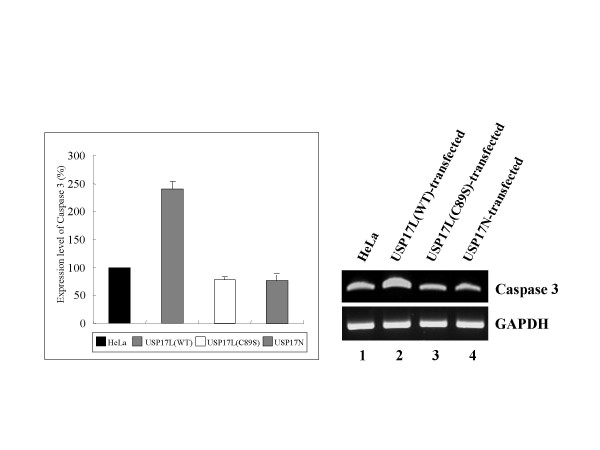
The effect of *USP17 *expression in HeLa cells. Relative amounts of *caspase 3 *produced by real-time PCR. The amount of *caspase 3 *DNA in the control group was calibrated as 100 and relative amounts of *caspase 3 *DNA in cells transfected with either *USP17L*, *USP17L *(C89S) or *USP17N *were determined.

Since it has been suggested that intracellular hyaluronan binding proteins are involved in tumor formation [32,33], we next investigated whether the USP17 subfamily proteins are involved in the regulation of cell viability and cell cycle for cancer cells. In order to investigate the cellular roles of USP17 subfamily proteins, myc-tagged *USP17L *was transfected into HEC-1A and HeLa cells to establish stable cell lines. However, we observed transfected cells undergoing apoptosis (Fig. 9) and approximately 3 weeks later, all cells died. However, the transfection of either *USP17L *(C89S) or *USP17N *into the same cell lines did not result in any sign of apoptosis (Fig. 9). This indicates that USP17 subfamily proteins containing hyaluronan binding motifs and their substrate(s) may regulate cell viability. Based on the observation of cell death for *USP17L*-transfected cells, we performed quantitative real-time PCR to compare fold change for the expression of caspase 3 as an indicator of apoptosis. The expression of caspase 3 showed more than two fold increase in *USP17L*-transfected cells compared with controls (Fig. 10). However, the expression of caspase 3 in *USP17L *(C89S)-transfected cells and in *USP17N*-transfected cells was similar to one in controls (Fig. 10). All reactions were performed triplicate.

**Figure 11:**
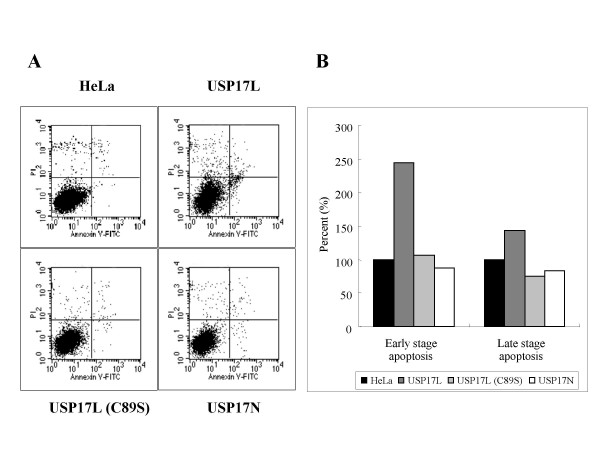
(A) FACS analysis. Cells expressing *USP17L*, *USP17L *(C89S), or *USP17N *were harvested 1 week apart for 3 weeks, fixed with ethanol, and stained with annexin V and PI. (B) The graph shows the ratio of cells in early (annexin V +, PI -) and late (annexin V +, PI +) stage apoptosis. The ratio of HeLa cells was calibrated as 100%.

In order to confirm the apoptosis induced by USP17 subfamily proteins, *USP17L *was stably expressed by the Tet-on system. The morphology of cells undergoing apoptosis was similar to that of ones transfected with pcDNA3-myc-*USP17L *(data not shown). Therefore, we harvested the *USP17L*-expressing cells at 1 week intervals for 3 weeks and analyzed through FACS analysis. The rate of apoptosis for cells expressing either *USP17L *or *USP17L *(CS) was analyzed by double-staining with annexin V and PI (Fig. 11). The cell stages of apoptosis were differentiated depending on the location in the quadrant (annexin V +, PI -; early stage) and (annexin V +, PI +; late stage) [34]. The *USP17L*-expressing cells by the Tet-on system undergoing the early stage apoptosis showed about 2.5 fold increase compared with control HeLa cells and those undergoing late stage apoptosis showed about 1.5 fold increase (Fig. 11). However, the significant change was not observed in cells expressing *USP17L *(CS) or *USP17N *(Fig. 11). This experiment was performed triplicate.

## Discussion

The ubiquitin-mediated protein degradation pathway has diverse functions involved in the regulation of protein activity, indicating the importance of the system in eukaryotic cells. Specific modulation by diverse deubiquitinating enzyme is a key process controlling many proteins associated with the ubiquitin-proteasome system. In this study, we isolated four novel members of *USP17 *subfamily and showed through sequence analysis that they have high degree of homology when compared with each other and *USP17 *(Additional File 1). Phylogenetic analysis for nucleotide sequences and deduced amino acid sequences of *USP17 *subfamily members revealed that all novel genes were grouped into the *USP17 *subfamily (Fig. 1). It has been recently reported that there are multiple homologous genomic sequences of *USP17 *subfamily, and one of them, *DUB-3*, encodes a cytokine-inducible deubiquitinating enzyme [21]. This is similar to murine *DUB *subfamily members including *DUB-1*, *DUB-2*, *DUB-2A*, and *DUB-1A *[1,9,25,26]. However, whether they have distinct functions or polymorphisms remains to be seen.

Since *USP17 *transcripts were expressed in various cancer cell lines and it has been suggested that intracellular hyaluronan binding proteins, which USP17 subfamily members belong to, are involved in tumor formation [33], we investigated whether USP17 subfamily proteins are involved in cell growth and proliferation. Interestingly, we observed that cancer cells (HeLa and HEC-1A) overexpressing USP17 subfamily proteins except USP17N appeared morphologically abnormal. In addition, FACS analysis showed that double-staining of USP17L-expressing, but not USP17N-expressing cells with annexin V and PI revealed increased apoptosis (Figs. 11A and 11B). These results suggest that USP17 subfamily proteins containing hyaluronan and RNA binding motifs are involved in signaling pathway of death for tumorigenic cells.

Besides the fact that the overexpression of USP17 subfamily proteins induces apoptosis in cancer cells, the ability to bind with hyaluronan is another important point used to elucidate the cellular functions of USP17 subfamily proteins. Hyaluronan is one of glycosaminoglycans and is believed to play various roles both in extracellular and pericellular matrices [32,35]. Since enriched hyaluronan is found in various tumor tissues [32,33], it was thought to be important for cancer. Several evidences have been proposed to prove the connection between hyaluronan and tumor growth. Therefore, hyaluronan is used as a marker for various cancers these days [32,33]. In addition, hyaluronan has been suggested that it is involved in the fundamental regulation of cell cycle, because hyaluronan is enriched in the nucleus during the cell division [31]. Since USP17 subfamily proteins except USP17N binds to hyaluronan and induces cell death, it is possible that hyaluronan and/or RNA binding domains of USP17 subfamily proteins may be involved in the regulation of cell cycle or tumorigenesis. Another possibility is that hyaluronan may serve as a bridge between the USP17 subfamily enzymes and their potential substrates, which are intracellular hyaluronan-binding proteins. Moreover, USP17 subfamily proteins are condensed at the nucleoli like hyaluronan dose, suggesting that USP17 subfamily proteins are involved in mRNA stability or chromatin remodeling similar to functions of PAI-RBP1 and Ki-1/57, which are hyaluronan/RNA binding motif-containing proteins [29,30]. Due to the fact that *in vivo *deubiquitinating enzyme assay for USP17 subfamily members showed the extensive reduction of ubiquitination for cellular proteins (Fig. 6), it is possible that a number of proteins including apoptosis-related proteins can be regulated by USP17 subfamily members. Important direction for future studies should include the isolation and characterization of substrates for USP17. This will help us to understand the cellular roles of USP17.

## Conclusion

In this study, we report that four novel USP17 subfamily members which encode a deubiquitinating enzyme have been identified and that the overexpression of these proteins leads to cell death. Our study demonstrates that USP17 subfamily members, which regulate apoptosis and cell death of cancerous cells, contain putative hyaluronan and RNA binding motifs. Despite of a decade of research since the discovery of DUB enzymes as an important molecule responsible for regulating protein degradation, much remains to be defined regarding their cellular functions. A better understanding of relation between USP17 subfamily proteins and cell viability will facilitate therapeutic strategies for regulation of cell proliferation.

## Methods

### Specimens and transfection

Chorionic villi samples used were described previously [36]. Various human cancer cells (HeLa, HEC-1A, NCCIT, and fibrosarcoma cells) were cultured in DMEM (Gibco BRL, Rockville, MD, USA) supplemented with 10% fetal bovine serum (Gibco BRL, Rockville, MD, USA). Transfection was performed with LipofectAMINE reagent (Gibco BRL, Rockville, MD, USA) according to manufacturer's instruction.

### cDNA synthesis and cloning for *USP17 *subfamily members

TRIzol reagent (Gibco BRL, Rockville, MD, USA) was used for total RNA isolation from obtained chorionic villi, HeLa, HEC-1A, NCCIT, and fibrosarcoma cells. cDNA synthesis from obtained total RNA was in accordance with the protocol of SuperScript Preamplification system (Gibco BRL, Rockville, MD, USA). The full-length cDNAs were amplified by RT-PCR in human chorionic villi tissues, HeLa, HEC-1A, NCCIT, and fibrosarcoma cells. Primers (Forward: 5'-CGGGATCCATGGAGGACG-3', Reverse: 5'-GGAATTCCACTGAGATCACT-3') used for isolating putative *DUB *genes are made on the basis of published sequences [GenBank: XM_252830]. Primers used were synthesized commercially and gel-purified prior to use (Bioneer, Daejeon, Korea). To express GST fusion proteins, pGEX-2TK-*USP17K*, pGEX-2TK-*USP17L*, pGEX-2TK-*USP17M*, and pGEX-2TK-*USP17N *were cloned by inserting each *USP17 *subfamily member into pGEX-2TK vector (Amersham Life Science, Uppsala, Sweden). BLAST programs on the web site established by the National Center of Biological Information [37] were used for similarity searches in the GenBank, and Pfam [27] program was used for prediction of possible motifs and domains in the protein sequences.

### Site-directed mutagenesis of *USP17 *subfamily members

Missense mutation constructs for pGEX-2TK-*USP17K *(C89S), pGEX-2TK-*USP17L *(C89S), pGEX-2TK-*USP17M *(C89S), and pGEX-2TK-*USP17N *(C89S) were derived from each wild type construct by PCR-based site-directed mutagenesis, using a QuikChange™ Site-Directed Mutagenesis kit (Stratagene, La Jolla, CA, USA) according to manufacturers' instructions. The two primers; forward primer (5'-GGGAAATACCCGCTACGTGAACGCTTC-3') and reverse primer (5'-GAAGCGTTCACGTAGCGGGTATTTCC-3') were used for replacing cysteine with serine at amino acid 89 of *USP17 *subfamily proteins. Primers used were synthesized commercially and gel-purified prior to use (Bioneer, Daejeon, Korea).

### Northern blot analysis

As described previously [36], Northern blot analysis was carried out to confirm the expression level of *USP17 *transcripts using a human tissue blot purchased from Clontech. The immobilized nucleic acids were hybridized with radiolabeled DNA probes. Probes used for this study was the 5' 500 bp and 3' 1 kb of *USP17L*. They were labeled by random primers with [^32^P]dCTP (Amersham Biosciences, Buckinghamshire, England). Hybridization was performed in a bag containing 5 × SSC, 1 × Denhardt's solution, 100 μg/ml of denatured salmon sperm DNA, 0.1% SDS and 50% formamide at 60°C and it was washed in 0.1 × SSC and 0.2% SDS, the membrane was exposed to Kodak X-ray films with an intensifying screen for 24 hours at -70°C.

### Deubiquitinating enzyme assays

Deubiquitinating enzyme assays were previously described [9]. For the *in vitro *deubiquitination assay, pGEX-2TK-*USP17K*, pGEX-2TK-*USP17K *(C89S), pGEX-2TK-*USP17L*, pGEX-2TK-*USP17L *(C89S), pGEX-2TK-*USP17M*, pGEX-2TK-*USP17M *(C89S), pGEX-2TK-*USP17N*, and pGEX-2TK-*USP17N *(C89S) were co-transformed into *E. coli *BL21 competent cells (Stratagene, La Jolla, CA, USA) with pACYC184-Ub-β-gal. Plasmid-bearing *E. coli *BL21 cells were grown in LB medium and induced with isopropyl-1-thio-β-D-galactopyranoside (IPTG) for 4 hours at 37°C. Cells were harvested and were lysed in cracking buffer (0.01 M phosphate [pH 7.4]. 8 M Urea, 1% SDS, and 1% β-mercaptoethanol). These whole cell lysates centrifuged for 10 min at 14,000 rpm and the supernatant was loaded onto a 7.5% SDS-PAGE gel. The immunoblotting was performed with a rabbit anti-β-gal antiserum (ICN, Costa Mesa, CA, USA) or a rabbit anti-GST antiserum (Upstate Biotechnology, Lake Placid, NY, USA).

The effect of *USP17 *expression on the ubiquitin-proteasome system *in vivo *was analyzed by transfecting pcDNA3-myc-*USP17L *or pcDNA3-myc-*USP17L *(C89S) along with pMT123-HA-ubiquitin into HEC-1A cells. After 24 hours of transfection, cells were harvested and lysed. 40 μg of total proteins was loaded in each lane of a 10% SDS-PAGE for immunoblotting analysis using an anti-HA antibody (Roche Applied Science, Mannheim, Germany). Equal loading was verified by immunoblotting against α-myc (Santa Cruz Biotechnology, Santa Cruz, CA, USA).

### Real-time PCR analysis and the Tet-on system

After the transfection of pcDNA3-myc-*USP17L*, the expression of caspase 3 as a marker for apoptosis was analyzed by real-time PCR (Opticon2, MJ Research, Ramsey, MN, USA). Product specificity was examined by melting curve analysis and agarose gel electrophoresis after each real-time PCR reaction. To normalize the expression data of the genes, GAPDH was used (Forward primer: 5'-ACTGGTGCTGCCAAGGCTGT-3', Reverse primer: 5'-TCCACCACCCTGTTGCTGTA-3'). Quantification was performed using an internal cDNA standard curve. Fold changes of caspase 3 expression in *USP17L*-overexpressed cells were calculated relative to the levels in control cells.

For the long-term (3 weeks) observation and FACS analysis, *USP17L *was expressed in HeLa cells using the Tet-on system (Gibco BRL, Rockville, MD, USA) by manufacturer's instruction. Expression of *USP17L *was confirmed by real-time PCR analysis.

### FACS analysis

For apoptosis analysis, HeLa-Tet-on cells expressing *USP17L *were harvested in PBS and resuspended 100 μl of binding buffer (10 mM Hepes/NaOH, pH 7.4, 140 mM NaCl, 2.5 mM CaCl_2_). 5 μl annexin V-FITC (BD Biosciences, Bedford, MA, USA) and 10 μl propidium iodide (PI, 50 μg/ml, BD Biosciences, Bedford, MA, USA) were added and incubated 15 minutes in the dark. Finally 400 μl of binding buffer was added and analyzed by FACSVantage™ SE system (BD Biosciences, Bedford, MA, USA). Cells in stage of apoptosis were differentiated depending on the location in quadrant (annexin V +, PI -; early stage) and (annexin V +, PI +; late stage) [34].

### Localization study

*USP17L *and *USP17N *were subcloned into pEGFP-C1 and pDsRed-C1 (Clontech, Palo Alto, CA, USA), respectively, and transfected into HeLa cells using ExGen500 (Fermentas, Hanover, MD, USA). After 48 hours, the localization of GFP-tagged *USP17L *and RFP-tagged *USP17N *was observed using a confocal microscopy (Radiance 2100 2Q, Bio-Rad, Hercules, CA, USA). In order to observe the morphology of nuclei for HeLa cells, DAPI staining was performed. After the fixation, 5 μl of DAPI staining solution (Roche Applied Science, Mannheim, Germany) was added in wells containing HeLa cells, which express either GFP-tagged *USP17L *or RFP-tagged *USP17N*. Stained cells were observed using Nikon Eclipse E600 microscopy.

### Hyaluronan- and RNA-binding assays

These assays were previously described [29]. 9 μg of pcDNA3-myc-*USP17L *or pcDNA3-myc-*USP17N *was transiently transfected into 1 × 10^6 ^HeLa cells in 100Φ dish by ExGen500 transfection reagent (Fermentas, Hanover, MD, USA). The supernatant from lysates was divided into 100 μl aliquots in Eppendorf tube and incubated at room temperature with 50 μg of hyaluronan (H5388, Sigma, St. Louis, MO, USA). For RNA binding assay, this procedure was repeated with using total RNA of HeLa cells instead of hyaluronan.

## Abbreviations

CPC, cetylpyridinium chloride; DUB, deubiquitinating enzyme; E1, ubiquitin-activating enzyme; E2, ubiquitin-conjugating enzyme; E3, ubiquitin ligase; ECL, enhanced chemiluminescence; GST, glutathione *S*-transferase; HA, hemagglutinin; IHABPS, intracellular hyaluronan binding proteins; IP, immunoprecipitation; JAMM, Jab1/Pab1/MPN-domain-containing metallo-enzyme; OTU, otu-domain ubiquitin aldehyde-binding protein; PCR, polymerase chain reaction; PI, propidium iodide; RHAMM, receptor form hyaluronan-mediated motility; SDS-PAGE; sodium dodecyl sulfate-polyacrylamide gel electrophoresis; Ub, ubiquitin; UBP, ubiquitin specific processing protease; UCH, ubiquitin C-terminal hydrolase.

## Authors' contributions

JMS participated in molecular cloning, sequence alignment and immunoassay. KJY carried out the molecular cloning and binding assays. MSK carried out the immunoassay and microscopic assay. DK carried out FACS analysis. KHB conceived of the study, participated in its design and coordination and helped to draft the manuscript. All authors read and approved the final manuscript.

## Supplementary Material

Additional File 1 Alignment of amino acid sequences for USP17, DUB-3, and novel USP17 subfamily members (USP17K to USP17N) (GenBank accession numbers: AY509884, BC100991, AF544011, AF544012, AY188990, and AY533200, respectively) using MegAlign software (Clustal method) from DNA Star (LaserGene). Different amino acids are blocked. Click here for file

## Figures and Tables

**Table 1:** Percent amino acid identity among USP17 subfamily and DUB subfamily members.

USP17A	USP17B	USP17C	USP17D	USP17F	USP17G	USP17H	USP17I	USP17J	USP17	DUB-3	USP17K	USP17L	USP17M	USP17N	
100.0	94.0	98.5	99.5	93.6	94.3	100.0	100.0	100.0	97.0	99.3	99.2	98.3	99.2	98.5	USP17A
	100.0	94.0	94.5	99.2	93.4	94.0	94.0	94.0	96.2	94.7	94.3	93.4	94.0	94.0	USP17B
		100.0	99.2	93.5	40.4	98.5	98.5	98.5	95.7	99.0	98.7	99.0	98.2	100.0	USP17C
			100.0	94.0	40.8	99.5	99.5	99.5	96.2	99.5	99.2	98.5	98.7	99.2	USP17D
				100.0	92.5	93.6	93.6	93.6	95.8	94.2	94.0	93.0	94.0	93.5	USP17F
					100.0	94.3	94.3	94.3	95.2	47.4	95.2	93.0	93.9	40.4	USP17G
						100.0	100.0	100.0	97.0	99.3	99.2	98.3	99.2	98.5	USP17H
							100.0	100.0	97.0	99.3	99.2	98.3	99.2	98.5	USP17I
								100.0	97.0	99.3	99.2	98.3	99.2	98.5	USP17J
									100.0	96.6	97.4	96.4	97.0	95.7	USP17
										100.0	99.5	98.8	99.0	99.0	DUB-3
											100.0	98.7	99.2	98.7	USP17K
												100.0	98.3	99.0	USP17L
													100.0	98.2	USP17M
														100.0	USP17N
