# Supplementary material for: Hyaluronan- and RNA-binding deubiquitinating enzymes of USP17 family members associated with cell viability
Source: BMC Genomics. 2006 Nov 16;7:292. doi: 10.1186/1471-2164-7-292 (PMC1665497; doi:10.1186/1471-2164-7-292)
Supplement: Additional File 1 — Alignment of amino acid sequences for USP17, DUB-3, and novel USP17 subfamily members (USP17K to USP17N) (GenBank accession numbers: AY509884, BC100991, AF544011, AF544012, AY188990, and AY533200, respectively) using MegAlign software (Clustal method) from DNA Star (LaserGene). Different amino acids are blocked. [file 1471-2164-7-292-S1.ppt]

## Slide 1
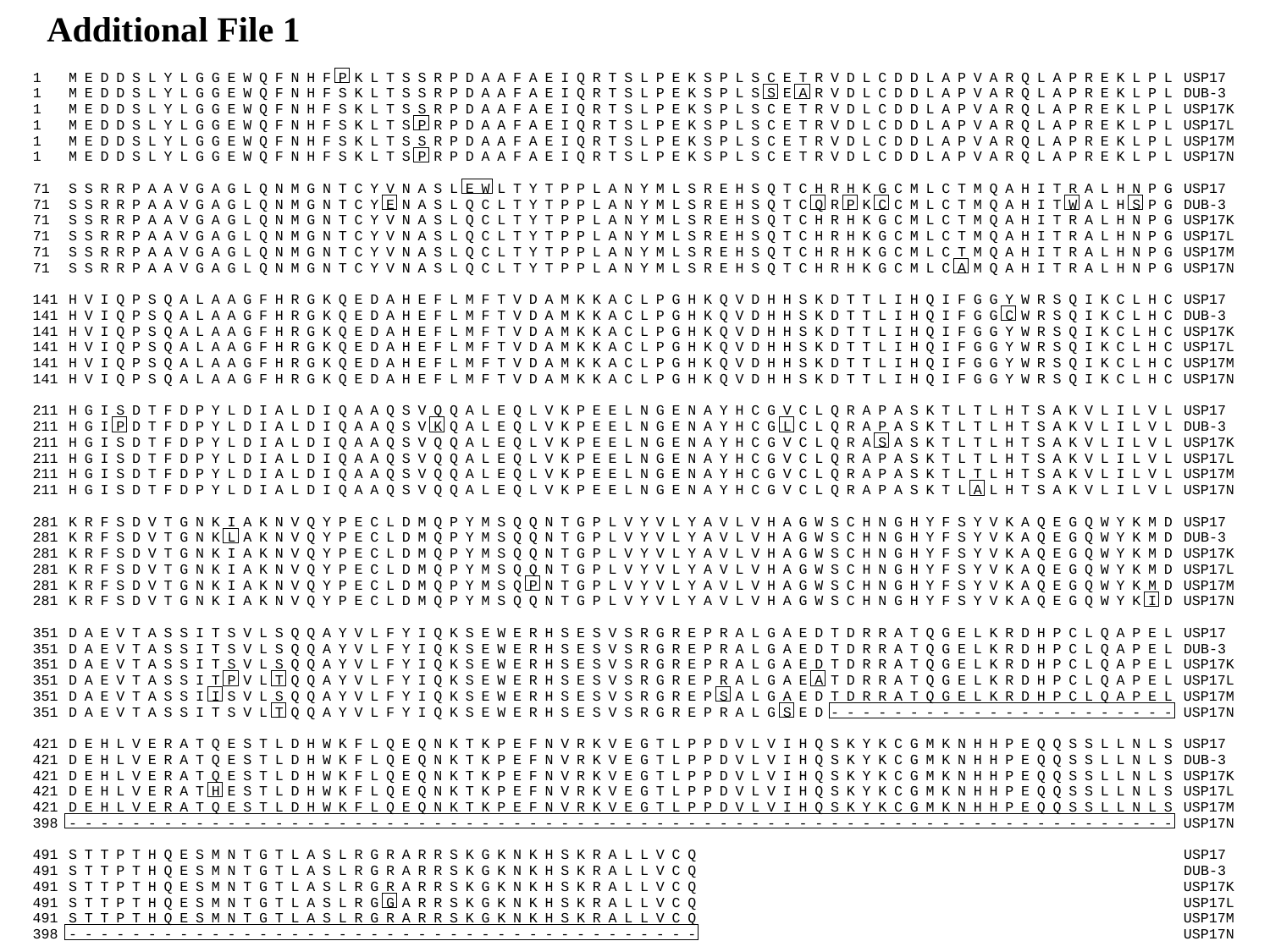

Additional File 1
1
M
E
D
D
S
L
Y
L
G
G
E
W
Q
F
N
H
F
P
K
L
T
S
S
R
P
D
A
A
F
A
E
I
Q
R
T
S
L
P
E
K
S
P
L
S
C
E
T
R
V
D
L
C
D
D
L
A
P
V
A
R
Q
L
A
P
R
E
K
L
P
L
USP17
1
M
E
D
D
S
L
Y
L
G
G
E
W
Q
F
N
H
F
S
K
L
T
S
S
R
P
D
A
A
F
A
E
I
Q
R
T
S
L
P
E
K
S
P
L
S
S
E
A
R
V
D
L
C
D
D
L
A
P
V
A
R
Q
L
A
P
R
E
K
L
P
L
DUB-3
1
M
E
D
D
S
L
Y
L
G
G
E
W
Q
F
N
H
F
S
K
L
T
S
S
R
P
D
A
A
F
A
E
I
Q
R
T
S
L
P
E
K
S
P
L
S
C
E
T
R
V
D
L
C
D
D
L
A
P
V
A
R
Q
L
A
P
R
E
K
L
P
L
USP17K
1
M
E
D
D
S
L
Y
L
G
G
E
W
Q
F
N
H
F
S
K
L
T
S
P
R
P
D
A
A
F
A
E
I
Q
R
T
S
L
P
E
K
S
P
L
S
C
E
T
R
V
D
L
C
D
D
L
A
P
V
A
R
Q
L
A
P
R
E
K
L
P
L
USP17L
1
M
E
D
D
S
L
Y
L
G
G
E
W
Q
F
N
H
F
S
K
L
T
S
S
R
P
D
A
A
F
A
E
I
Q
R
T
S
L
P
E
K
S
P
L
S
C
E
T
R
V
D
L
C
D
D
L
A
P
V
A
R
Q
L
A
P
R
E
K
L
P
L
USP17M
1
M
E
D
D
S
L
Y
L
G
G
E
W
Q
F
N
H
F
S
K
L
T
S
P
R
P
D
A
A
F
A
E
I
Q
R
T
S
L
P
E
K
S
P
L
S
C
E
T
R
V
D
L
C
D
D
L
A
P
V
A
R
Q
L
A
P
R
E
K
L
P
L
USP17N
71
S
S
R
R
P
A
A
V
G
A
G
L
Q
N
M
G
N
T
C
Y
V
N
A
S
L
E
W
L
T
Y
T
P
P
L
A
N
Y
M
L
S
R
E
H
S
Q
T
C
H
R
H
K
G
C
M
L
C
T
M
Q
A
H
I
T
R
A
L
H
N
P
G
USP17
71
S
S
R
R
P
A
A
V
G
A
G
L
Q
N
M
G
N
T
C
Y
E
N
A
S
L
Q
C
L
T
Y
T
P
P
L
A
N
Y
M
L
S
R
E
H
S
Q
T
C
Q
R
P
K
C
C
M
L
C
T
M
Q
A
H
I
T
W
A
L
H
S
P
G
DUB-3
71
S
S
R
R
P
A
A
V
G
A
G
L
Q
N
M
G
N
T
C
Y
V
N
A
S
L
Q
C
L
T
Y
T
P
P
L
A
N
Y
M
L
S
R
E
H
S
Q
T
C
H
R
H
K
G
C
M
L
C
T
M
Q
A
H
I
T
R
A
L
H
N
P
G
USP17K
71
S
S
R
R
P
A
A
V
G
A
G
L
Q
N
M
G
N
T
C
Y
V
N
A
S
L
Q
C
L
T
Y
T
P
P
L
A
N
Y
M
L
S
R
E
H
S
Q
T
C
H
R
H
K
G
C
M
L
C
T
M
Q
A
H
I
T
R
A
L
H
N
P
G
USP17L
71
S
S
R
R
P
A
A
V
G
A
G
L
Q
N
M
G
N
T
C
Y
V
N
A
S
L
Q
C
L
T
Y
T
P
P
L
A
N
Y
M
L
S
R
E
H
S
Q
T
C
H
R
H
K
G
C
M
L
C
T
M
Q
A
H
I
T
R
A
L
H
N
P
G
USP17M
71
S
S
R
R
P
A
A
V
G
A
G
L
Q
N
M
G
N
T
C
Y
V
N
A
S
L
Q
C
L
T
Y
T
P
P
L
A
N
Y
M
L
S
R
E
H
S
Q
T
C
H
R
H
K
G
C
M
L
C
A
M
Q
A
H
I
T
R
A
L
H
N
P
G
USP17N
141
H
V
I
Q
P
S
Q
A
L
A
A
G
F
H
R
G
K
Q
E
D
A
H
E
F
L
M
F
T
V
D
A
M
K
K
A
C
L
P
G
H
K
Q
V
D
H
H
S
K
D
T
T
L
I
H
Q
I
F
G
G
Y
W
R
S
Q
I
K
C
L
H
C
USP17
141
H
V
I
Q
P
S
Q
A
L
A
A
G
F
H
R
G
K
Q
E
D
A
H
E
F
L
M
F
T
V
D
A
M
K
K
A
C
L
P
G
H
K
Q
V
D
H
H
S
K
D
T
T
L
I
H
Q
I
F
G
G
C
W
R
S
Q
I
K
C
L
H
C
DUB-3
141
H
V
I
Q
P
S
Q
A
L
A
A
G
F
H
R
G
K
Q
E
D
A
H
E
F
L
M
F
T
V
D
A
M
K
K
A
C
L
P
G
H
K
Q
V
D
H
H
S
K
D
T
T
L
I
H
Q
I
F
G
G
Y
W
R
S
Q
I
K
C
L
H
C
USP17K
141
H
V
I
Q
P
S
Q
A
L
A
A
G
F
H
R
G
K
Q
E
D
A
H
E
F
L
M
F
T
V
D
A
M
K
K
A
C
L
P
G
H
K
Q
V
D
H
H
S
K
D
T
T
L
I
H
Q
I
F
G
G
Y
W
R
S
Q
I
K
C
L
H
C
USP17L
141
H
V
I
Q
P
S
Q
A
L
A
A
G
F
H
R
G
K
Q
E
D
A
H
E
F
L
M
F
T
V
D
A
M
K
K
A
C
L
P
G
H
K
Q
V
D
H
H
S
K
D
T
T
L
I
H
Q
I
F
G
G
Y
W
R
S
Q
I
K
C
L
H
C
USP17M
141
H
V
I
Q
P
S
Q
A
L
A
A
G
F
H
R
G
K
Q
E
D
A
H
E
F
L
M
F
T
V
D
A
M
K
K
A
C
L
P
G
H
K
Q
V
D
H
H
S
K
D
T
T
L
I
H
Q
I
F
G
G
Y
W
R
S
Q
I
K
C
L
H
C
USP17N
211
H
G
I
S
D
T
F
D
P
Y
L
D
I
A
L
D
I
Q
A
A
Q
S
V
Q
Q
A
L
E
Q
L
V
K
P
E
E
L
N
G
E
N
A
Y
H
C
G
V
C
L
Q
R
A
P
A
S
K
T
L
T
L
H
T
S
A
K
V
L
I
L
V
L
USP17
211
H
G
I
P
D
T
F
D
P
Y
L
D
I
A
L
D
I
Q
A
A
Q
S
V
K
Q
A
L
E
Q
L
V
K
P
E
E
L
N
G
E
N
A
Y
H
C
G
L
C
L
Q
R
A
P
A
S
K
T
L
T
L
H
T
S
A
K
V
L
I
L
V
L
DUB-3
211
H
G
I
S
D
T
F
D
P
Y
L
D
I
A
L
D
I
Q
A
A
Q
S
V
Q
Q
A
L
E
Q
L
V
K
P
E
E
L
N
G
E
N
A
Y
H
C
G
V
C
L
Q
R
A
S
A
S
K
T
L
T
L
H
T
S
A
K
V
L
I
L
V
L
USP17K
211
H
G
I
S
D
T
F
D
P
Y
L
D
I
A
L
D
I
Q
A
A
Q
S
V
Q
Q
A
L
E
Q
L
V
K
P
E
E
L
N
G
E
N
A
Y
H
C
G
V
C
L
Q
R
A
P
A
S
K
T
L
T
L
H
T
S
A
K
V
L
I
L
V
L
USP17L
211
H
G
I
S
D
T
F
D
P
Y
L
D
I
A
L
D
I
Q
A
A
Q
S
V
Q
Q
A
L
E
Q
L
V
K
P
E
E
L
N
G
E
N
A
Y
H
C
G
V
C
L
Q
R
A
P
A
S
K
T
L
T
L
H
T
S
A
K
V
L
I
L
V
L
USP17M
211
H
G
I
S
D
T
F
D
P
Y
L
D
I
A
L
D
I
Q
A
A
Q
S
V
Q
Q
A
L
E
Q
L
V
K
P
E
E
L
N
G
E
N
A
Y
H
C
G
V
C
L
Q
R
A
P
A
S
K
T
L
A
L
H
T
S
A
K
V
L
I
L
V
L
USP17N
281
K
R
F
S
D
V
T
G
N
K
I
A
K
N
V
Q
Y
P
E
C
L
D
M
Q
P
Y
M
S
Q
Q
N
T
G
P
L
V
Y
V
L
Y
A
V
L
V
H
A
G
W
S
C
H
N
G
H
Y
F
S
Y
V
K
A
Q
E
G
Q
W
Y
K
M
D
USP17
281
K
R
F
S
D
V
T
G
N
K
L
A
K
N
V
Q
Y
P
E
C
L
D
M
Q
P
Y
M
S
Q
Q
N
T
G
P
L
V
Y
V
L
Y
A
V
L
V
H
A
G
W
S
C
H
N
G
H
Y
F
S
Y
V
K
A
Q
E
G
Q
W
Y
K
M
D
DUB-3
281
K
R
F
S
D
V
T
G
N
K
I
A
K
N
V
Q
Y
P
E
C
L
D
M
Q
P
Y
M
S
Q
Q
N
T
G
P
L
V
Y
V
L
Y
A
V
L
V
H
A
G
W
S
C
H
N
G
H
Y
F
S
Y
V
K
A
Q
E
G
Q
W
Y
K
M
D
USP17K
281
K
R
F
S
D
V
T
G
N
K
I
A
K
N
V
Q
Y
P
E
C
L
D
M
Q
P
Y
M
S
Q
Q
N
T
G
P
L
V
Y
V
L
Y
A
V
L
V
H
A
G
W
S
C
H
N
G
H
Y
F
S
Y
V
K
A
Q
E
G
Q
W
Y
K
M
D
USP17L
281
K
R
F
S
D
V
T
G
N
K
I
A
K
N
V
Q
Y
P
E
C
L
D
M
Q
P
Y
M
S
Q
P
N
T
G
P
L
V
Y
V
L
Y
A
V
L
V
H
A
G
W
S
C
H
N
G
H
Y
F
S
Y
V
K
A
Q
E
G
Q
W
Y
K
M
D
USP17M
281
K
R
F
S
D
V
T
G
N
K
I
A
K
N
V
Q
Y
P
E
C
L
D
M
Q
P
Y
M
S
Q
Q
N
T
G
P
L
V
Y
V
L
Y
A
V
L
V
H
A
G
W
S
C
H
N
G
H
Y
F
S
Y
V
K
A
Q
E
G
Q
W
Y
K
I
D
USP17N
351
D
A
E
V
T
A
S
S
I
T
S
V
L
S
Q
Q
A
Y
V
L
F
Y
I
Q
K
S
E
W
E
R
H
S
E
S
V
S
R
G
R
E
P
R
A
L
G
A
E
D
T
D
R
R
A
T
Q
G
E
L
K
R
D
H
P
C
L
Q
A
P
E
L
USP17
351
D
A
E
V
T
A
S
S
I
T
S
V
L
S
Q
Q
A
Y
V
L
F
Y
I
Q
K
S
E
W
E
R
H
S
E
S
V
S
R
G
R
E
P
R
A
L
G
A
E
D
T
D
R
R
A
T
Q
G
E
L
K
R
D
H
P
C
L
Q
A
P
E
L
DUB-3
351
D
A
E
V
T
A
S
S
I
T
S
V
L
S
Q
Q
A
Y
V
L
F
Y
I
Q
K
S
E
W
E
R
H
S
E
S
V
S
R
G
R
E
P
R
A
L
G
A
E
D
T
D
R
R
A
T
Q
G
E
L
K
R
D
H
P
C
L
Q
A
P
E
L
USP17K
351
D
A
E
V
T
A
S
S
I
T
P
V
L
T
Q
Q
A
Y
V
L
F
Y
I
Q
K
S
E
W
E
R
H
S
E
S
V
S
R
G
R
E
P
R
A
L
G
A
E
A
T
D
R
R
A
T
Q
G
E
L
K
R
D
H
P
C
L
Q
A
P
E
L
USP17L
351
D
A
E
V
T
A
S
S
I
I
S
V
L
S
Q
Q
A
Y
V
L
F
Y
I
Q
K
S
E
W
E
R
H
S
E
S
V
S
R
G
R
E
P
S
A
L
G
A
E
D
T
D
R
R
A
T
Q
G
E
L
K
R
D
H
P
C
L
Q
A
P
E
L
USP17M
351
D
A
E
V
T
A
S
S
I
T
S
V
L
T
Q
Q
A
Y
V
L
F
Y
I
Q
K
S
E
W
E
R
H
S
E
S
V
S
R
G
R
E
P
R
A
L
G
S
E
D
-
-
-
-
-
-
-
-
-
-
-
-
-
-
-
-
-
-
-
-
-
-
USP17N
421
D
E
H
L
V
E
R
A
T
Q
E
S
T
L
D
H
W
K
F
L
Q
E
Q
N
K
T
K
P
E
F
N
V
R
K
V
E
G
T
L
P
P
D
V
L
V
I
H
Q
S
K
Y
K
C
G
M
K
N
H
H
P
E
Q
Q
S
S
L
L
N
L
S
USP17
421
D
E
H
L
V
E
R
A
T
Q
E
S
T
L
D
H
W
K
F
L
Q
E
Q
N
K
T
K
P
E
F
N
V
R
K
V
E
G
T
L
P
P
D
V
L
V
I
H
Q
S
K
Y
K
C
G
M
K
N
H
H
P
E
Q
Q
S
S
L
L
N
L
S
DUB-3
421
D
E
H
L
V
E
R
A
T
Q
E
S
T
L
D
H
W
K
F
L
Q
E
Q
N
K
T
K
P
E
F
N
V
R
K
V
E
G
T
L
P
P
D
V
L
V
I
H
Q
S
K
Y
K
C
G
M
K
N
H
H
P
E
Q
Q
S
S
L
L
N
L
S
USP17K
421
D
E
H
L
V
E
R
A
T
H
E
S
T
L
D
H
W
K
F
L
Q
E
Q
N
K
T
K
P
E
F
N
V
R
K
V
E
G
T
L
P
P
D
V
L
V
I
H
Q
S
K
Y
K
C
G
M
K
N
H
H
P
E
Q
Q
S
S
L
L
N
L
S
USP17L
421
D
E
H
L
V
E
R
A
T
Q
E
S
T
L
D
H
W
K
F
L
Q
E
Q
N
K
T
K
P
E
F
N
V
R
K
V
E
G
T
L
P
P
D
V
L
V
I
H
Q
S
K
Y
K
C
G
M
K
N
H
H
P
E
Q
Q
S
S
L
L
N
L
S
USP17M
398
-
-
-
-
-
-
-
-
-
-
-
-
-
-
-
-
-
-
-
-
-
-
-
-
-
-
-
-
-
-
-
-
-
-
-
-
-
-
-
-
-
-
-
-
-
-
-
-
-
-
-
-
-
-
-
-
-
-
-
-
-
-
-
-
-
-
-
-
-
-
USP17N
491
S
T
T
P
T
H
Q
E
S
M
N
T
G
T
L
A
S
L
R
G
R
A
R
R
S
K
G
K
N
K
H
S
K
R
A
L
L
V
C
Q
USP17
491
S
T
T
P
T
H
Q
E
S
M
N
T
G
T
L
A
S
L
R
G
R
A
R
R
S
K
G
K
N
K
H
S
K
R
A
L
L
V
C
Q
DUB-3
491
S
T
T
P
T
H
Q
E
S
M
N
T
G
T
L
A
S
L
R
G
R
A
R
R
S
K
G
K
N
K
H
S
K
R
A
L
L
V
C
Q
USP17K
491
S
T
T
P
T
H
Q
E
S
M
N
T
G
T
L
A
S
L
R
G
G
A
R
R
S
K
G
K
N
K
H
S
K
R
A
L
L
V
C
Q
USP17L
491
S
T
T
P
T
H
Q
E
S
M
N
T
G
T
L
A
S
L
R
G
R
A
R
R
S
K
G
K
N
K
H
S
K
R
A
L
L
V
C
Q
USP17M
398
-
-
-
-
-
-
-
-
-
-
-
-
-
-
-
-
-
-
-
-
-
-
-
-
-
-
-
-
-
-
-
-
-
-
-
-
-
-
-
-
USP17N
